# Perinatal outcomes after induced termination of pregnancy by methods: A nationwide register-based study of first births in Finland 1996–2013

**DOI:** 10.1371/journal.pone.0184078

**Published:** 2017-09-01

**Authors:** Situ KC, Elina Hemminki, Mika Gissler, Suvi M. Virtanen, Reija Klemetti

**Affiliations:** 1 School of Social Sciences, University of Tampere, Tampere, Finland; 2 Department of Health and Social Care Systems, National Institute for Health and Welfare, Helsinki, Finland; 3 Department of Information Services, National Institute for Health and Welfare, Helsinki, Finland; 4 Department of Neurobiology, Care Sciences and Society, Karolinska Institute, Stockholm, Sweden; 5 Department of Public Health Solutions, National Institute for Health and Welfare, Helsinki, Finland; 6 Department of Welfare, National Institute for Health and Welfare, Helsinki, Finland; VU medisch centrum, NETHERLANDS

## Abstract

**Background:**

Women with previous terminations of pregnancy (TOPs) before their first birth have been associated with poorer perinatal outcomes. However, previous studies on the perinatal outcomes by the method in previous TOPs are inconsistent.

**Objective:**

To examine the perinatal outcomes of the first-time mothers with singleton births, by the method of previous TOP (medical and surgical vs no TOP, and surgical vs medical).

**Method:**

This is a nationwide register-based study including 419,879 first-time Finnish mothers with singleton birth during the time period 1996–2013. Mothers having their first birth were identified from the Medical Birth Register and linked to the Abortion Register by their identification numbers. Multinomial logistic regression analysis was performed to examine the risk for preterm birth, low birth weight, small for gestational age and perinatal death by the method in previous TOPs.

**Results:**

Among the first-time mothers, 87.0% had no history of TOPs, 3.2% had a history of medical TOP(s), 9.2% had a history of surgical TOP(s) and 0.6% had a history of both (medical and surgical) TOP(s). No significant differences in perinatal outcomes were found among the women with surgical TOPs, compared to the women with no TOPs. In unadjusted analysis, increased odds for preterm birth and low birth weight were found when comparing women having previous surgical TOPs with medical TOPs. Even after the adjustment of potential confounders, odds for preterm birth < 37 weeks (OR = 1.19, 95% CI = 1.04–1.36) and low birth weight < 2500 g (OR = 1.16, 95% CI = 1.00–1.35) remained significant. After restricting data to the single TOP, the results were similar; OR for both preterm birth and low birth weight was 1.18 (95% CIs = 1.02–1.36 and 1.01–1.38).

**Conclusion:**

Perinatal outcomes did not differ among the mothers with surgical TOPs compared to the mothers with no TOPs, while the outcomes were poorer after surgical TOP(s) than after medical TOP(s).

## Introduction

In Europe, termination of pregnancies (subsequently TOPs) are common, and in Western European countries most TOPs are performed before the first birth [1]. Finland has low rate of TOP, and in 2014 the rate was 8.5 per 1,000 women aged 15–49 years old. The highest rate was among women aged 20–24 years old (16.8 per 1,000 women), which is well below the mean age (28.6 years old) of a woman’s first childbirth [2].

A termination of pregnancy can be performed by surgical (dilatation and uterine evacuation) or medical (antiprogestin mifepristone and misoprostol) methods. In Europe, medical TOPs began in France in 1998 [3]. Mifepristone received authorization in Finland in 2000. Since then, there have been increased use of medical termination of pregnancy and it was nearly 90% in 2014 [2].

Whether or not an induced termination of pregnancy prior to the first birth adversely influences the outcome of that birth has been previously debated [4–8]. There are evidences of an increased risk of preterm birth with many TOPs prior to the first birth [5–8], but these results refer to the time period when most TOPs were surgical. However, some studies did not found an association between previous TOPs and preterm birth/ low birth weight [4,9–11].

Few studies have considered the method of TOPs, with regard to the outcomes in subsequent birth [9,12–18]. Some studies have reported a higher risk of preterm birth and low birth weight after surgical TOPs, when compared to medical TOPs [15,17], but others have found no increased risk in outcomes between these methods [14,16,18]. Although no other studies have taken into account the number of TOPs when comparing these methods, a study from China [15] has reported an increased risk for preterm birth among those mothers with repeated surgical TOPs, compared to those mothers with repeated medical TOPs.

A previous study from Finland found an increased risk for poorer perinatal outcomes after many TOPs, however data was too scant to study the outcomes by the method of abortion [8]. Thus the purpose of this study was to examine the perinatal outcomes of first-time mothers with singleton birth by the method of TOP: medical and surgical vs no TOP, and surgical vs. medical TOP(s), while adjusting for confounding factors. Additionally, comparisons were made between those mothers with only one previous TOP in their reproductive histories.

## Methods

The study was approved by the ethical committee of National Institute for Health and Welfare (THL). A positive statement from THL ethics committee (22.10.2009), a positive statement with regard to the amendment of the data, and a permission to use the data were received from THL (25.10.2014). The information used in this study were anonymized prior the analysis.

In this population-based cohort study, we used the nationwide Medical Birth Register (MBR) and Abortion Register (AR), which were maintained by the National Institute for Health and Welfare (THL). All the mothers having had their first birth during the time period ranging from 1996 to 2013 were identified from the MBR, and these mothers were linked to the AR to determine the TOPs (1983–2013) they had prior to their first birth. The MBR was started in 1987, and it contains information about each mother’s background characteristics, care during pregnancy and delivery, and newborn care up to 7 days of age [2]. The AR has been functional since 1950, and computerized data are available since 1983 [19]. The register contains information on a woman’s background, gestational age, indication for TOPs, dates, procedures and complications occurring during the process [19]. Overall, the information in both registers is relatively complete, and the data quality is high [19–20].

For this study, the mothers were divided into four study groups by their TOP histories and methods: no prior TOP, medical TOPs only, surgical TOPs only and both types of TOPs. The medical TOPs included TOPs performed using mifepristone alone, or in combination with misoprostol. Surgical TOPs included TOPs performed using either dilatation and curettage or vacuum aspiration. Mothers who had undergone multiple TOPs using both medical and surgical methods were included in both types of TOPs. Only the mothers with successful TOPs were included. The proportion of failed TOPs is very low; 0.4% in 2009–2015 according to the Abortion Register.

The outcome measures, gestational age at birth, birth weight, small for gestational age and perinatal death were retrieved from the MBR. The gestational age at birth in the MBR is the clinicians’ best estimate at birth, based on ultrasound examination(s) and the date of last menstruation. The birth was defined as preterm if the gestational age at birth was less than 37 weeks, very preterm if the gestational age at birth was less than 32 weeks and extremely preterm if the gestational age was less than 28 weeks. Birth weights of less than 2,500 grams and 1,500 grams were defined as low birth weight and very low birth weight, respectively. Small for gestational age (SGA) was defined according to sex-specific Finnish standards for newborn infants between 24 and 43 gestation weeks [21]. Perinatal deaths referred to stillbirths from 22 weeks of gestation and early neonatal deaths until the end of the first week after birth.

The background characteristics of women were received from the MBR and they refer to the time of the birth of the baby. The urbanity of the maternal municipality of residence was categorized according to Statistics Finland, and the categories were further grouped into urban, semi-urban, rural and abroad. In the MBR, marital status of mothers was categorized into seven categories; married and living together with spouse, registered partnership, married and living separated from spouse, never married, divorced, widowed and unknown. These were further categorized into three groups; married/cohabiting, unmarried/single and unknown. In this study, we treated all variables as categorical variables. Information concerning socioeconomic status of the mothers was incomplete. So, maternal smoking and urbanity of municipality were used to explain the socioeconomic status of mothers.

The statistical software, SPSS 23, was used for the analysis. Cross tabulations based on the study groups were calculated and chi-square test were used to study statistical significance. The level of statistical significance was set at p<0.05. Those mothers with previous surgical TOPs and those with previous medical TOPs were separately compared to women with no previous TOP, adjusting for differences in their background characteristics using a multivariate logistic regression (odds ratio and 95% confidence interval). The potential confounders were selected on the basis of the previous literature on the maternal risk factors of birth outcomes, and their availability and quality in the registers. Mothers with previous TOPs (surgical or medical) were compared to those without previous TOP, after adjusting for the maternal age, marital status, urbanity of the municipality of residence, history of smoking during pregnancy and the year of childbirth.

Mothers with previous surgical TOPs were compared to those with previous medical TOPs, after adjusting for the maternal age, marital status, urbanity of the municipality of residence, history of smoking during pregnancy and the year of childbirth. In the second model, additional adjustments (number of previous TOPs, gestational age at TOP and year of last TOP) were added. Since the number of women having had both medical and surgical TOPs was small when compared to the other groups, these mothers were excluded from the regression analysis. Lastly, a sub-analysis for those mothers having had only one previous surgical or medical TOP was conducted, after adjusting for the same confounders as above.

## Results

A total of 419,879 first-time mothers having had singleton birth from 1996–2013 were identified from the MBR. According to the AR, 365,356 (87.0%) of the mothers had no history of TOP, 13,450 (3.2%) had histories of medical TOP(s), 38,659 (9.2%) had histories of surgical TOP(s) and 2,414 (0.6%) had histories of both medical and surgical TOP(s). The background characteristics differed with regard to several aspects between these subgroups (Table 1). The mothers with histories of previous TOPs were more often younger, single, urban residents and smokers than the mothers without previous TOP. When compared to the mothers with previous medical TOPs, the mothers with previous surgical TOPs had more repeated TOPs, had their last TOP in earlier years, and the time difference between their first birth and last TOP was longer (Table 2).

**Table 1:** Background characteristics of Finnish first-time mothers in 1996–2013 by previous TOPs and methods.

Characteristics	No prior TOP (n = 365356)	Medical TOP (n = 13450)	Surgical TOP (n = 38659)	Both (n = 2414)	Total (n = 419879)	P-value*
	%	%	%	%	%	
Maternal age						
Mean (SD)	27.4 (5.2)	26.1 (5.3)	28.2 (5.5)	27.4 (5.5)	27.4 (5.2)	<0.0001
≤19	5.8	8.3	4.6	3.6	5.8	
20–24	24.5	35.7	22.6	28.1	24.7	
25–29	36.9	31.4	32.7	32.6	36.3	
30–34	23.6	17.2	26.3	23.2	23.7	
35–39	7.7	5.9	11.2	9.6	7.9	
≥40	1.5	1.4	2.6	2.9	1.6	
Marital status						
Married/cohabiting	86.0	78.6	77.5	74.4	84.9	<0.0001
Unmarried/Single	12.6	20.8	20.3	25.1	13.7	
Unknown	1.4	0.6	2.2	0.6	1.4	
Type of residence						
Urban	71.1	73.3	73.3	76.0	71.4	<0.0001
Semi-urban	14.2	14.1	14.1	12.2	14.1	
Rural	14.1	11.9	13.1	11.2	13.9	
Abroad	0.6	0.7	0.4	0.5	0.6	
Smoking status						
No smoking	82.7	63.5	67.6	54.8	80.5	<0.0001
Stopped smoking in third trimester	4.8	11.9	6.3	11.6	5.2	
Smoked after first trimester	10.5	22.6	23.8	31.5	12.3	
Birth year of child						
1996–2000	26.7	3.9	36.1	7.9	26.7	<0.0001
2001–2005	27.2	12.4	33.2	18.5	27.2	
2006–2010	29.1	43.9	22.9	45.3	29.1	
2011–2013	17.0	39.8	7.9	28.3	16.9	

*P-value from chi square test and statistical significance at the level of 0.05

**Table 2:** History of TOPs of Finnish first-time mothers in 1996–2013 by their methods.

History of TOPs	Medical TOP (n = 13450)	Surgical TOP (n = 38659)	Both TOP (n = 2414)	Total (n = 54523)	P-value*
	%	%	%	%	
Number of TOPs					
1	90.6	87.5	0.0	84.4	<0.0001
2	8.1	10.5	71.3	12.6	
≥3	1.3	2.0	28.7	3.0	
Gestational age at TOP					
<12 weeks	77.0	90.4	61.4	85.8	<0.0001
≥12 weeks	23.0	9.6	38.6	14.2	
Year of last TOP					
1987–1994	3.3	33.1	4.9	23.9	<0.0001
1995–1998	3.3	29.4	7.2	21.4	
1999–2003	25.0	28.2	28.9	27.4	
2004–2013	68.3	9.2	59.1	27.3	
Difference between first birth and last TOP					
5–38 months	50.2	24.6	53.4	32.8	<0.0001
39–81 months	35.4	33.0	34.0	33.7	
82–310 months	14.4	42.4	12.6	33.5	

*P-value from chi square test and statistical significance at the level of 0.05

When compared to those mothers with no previous TOP, the perinatal outcomes were poorer among those mothers with previous surgical or both types of TOPs, but not among the mothers with previous medical TOPs only (Table 3). The incidence of preterm birth was lower among the mothers with previous medical TOPs, when compared to those mothers without previous TOPs.

**Table 3:** Incidence of perinatal outcomes among the Finnish first-time mothers in 1996–2013 by previous TOPs and methods.

Perinatal outcomes	No prior TOP (n = 365356)		Medical TOP (n = 13450)		Surgical TOP (n = 38659)		Both (n = 2414)		Total (n = 419879)	
	n	/1000	n	/1000	n	/1000	n	/1000	n	/1000
Extremely preterm birth <28 weeks	1122	3	38	3	154	4	11	5	1325	3
Very preterm birth <32 weeks	1843	5	57	4	232	6	10	4	2142	5
Preterm birth <37 weeks	17041	47	515	38	1882	49	105	44	19542	47
Very low birth weight <1500 grams	2805	8	90	7	341	9	20	8	3256	8
Low birth weight <2500 grams	12439	34	417	31	1447	37	88	36	14391	34
Small for gestational age	17897	49	687	51	2047	53	136	56	20767	49
Perinatal death	1622	4	58	4	206	5	13	5	1899	5

The unadjusted logistic regression analysis showed increased risk for all types of preterm birth and low birth weight after surgical TOPs and decreased risk for all studied perinatal outcomes after medical TOPs, when compared to the mothers without previous TOP (Table 4). Compared to the mothers with previous medical TOPs, the mothers with previous surgical TOPs had increased risk for all studied outcomes, with the exception of SGA and perinatal death.

**Table 4:** Crude and adjusted ORs and 95% confidence intervals for perinatal outcomes of Finnish first-time mothers according to the method of TOP.

Perinatal outcomes	Odds Ratios and 95% Confidence Interval							
	Medical vs no TOP^1^		Surgical vs no TOP^1^		Surgical vs medical TOP^2^		Surgical vs medical TOP, only one TOP^3^	
	OR	95% CI	OR	95% CI	OR	95% CI	OR	95% CI
Extremely preterm birth <28 weeks								
Crude Model	0.91	0.66–1.26	1.30	1.10–1.54	1.43	0.99–2.04	1.32	0.91–1.92
Adjusted Model I	0.94	0.68–1.30	1.16	0.98–1.38	1.20	0.79–1.81	1.12	0.73–1.73
Adjusted Model II					1.43	0.90–2.30	1.39	0.84–2.30
Very preterm birth <32 weeks								
Crude Model	0.83	0.64–1.08	1.19	1.04–1.37	1.44	1.07–1.92	1.33	0.98–1.80
Adjusted Model I	0.88	0.67–1.15	1.03	0.89–1.18	1.23	0.87–1.71	1.13	0.80–1.61
Adjusted Model II					1.24	0.85–1.81	1.23	0.83–1.84
Preterm births <37 weeks								
Crude Model	0.81	0.74–0.89	1.05	1.00–1.10	1.29	1.17–1.43	1.26	1.13–1.40
Adjusted Model I	0.83	0.76–0.91	1.00	0.95–1.05	1.16	1.03–1.30	1.15	1.02–1.29
Adjusted Model II					1.19	1.04–1.36	1.18	1.02–1.36
Very low birth weight <1500 grams								
Crude Model	0.87	0.70–1.07	1.15	1.03–1.29	1.33	1.05–1.68	1.25	0.98–1.60
Adjusted Model I	0.88	0.71–1.10	1.00	0.89–1.12	1.16	0.89–1.52	1.11	0.84–1.47
Adjusted Model II					1.27	0.93–1.73	1.27	0.91–1.76
Low birth weight <2500 grams								
Crude Model	0.91	0.82–1.00	1.10	1.04–1.17	1.22	1.09–1.36	1.21	1.08–1.36
Adjusted Model I	0.86	0.78–0.95	0.98	0.93–1.04	1.16	1.02–1.32	1.16	1.01–1.32
Adjusted Model II					1.16	1.00–1.35	1.18	1.01–1.38
Small for gestational age								
Crude Model	0.96	0.88–1.03	0.92	0.88–0.97	0.96	0.88–1.05	0.96	0.88–1.05
Adjusted Model I	1.05	0.97–1.14	1.07	1.02–1.12	0.99	0.89–1.10	0.99	0.89–1.10
Adjusted Model II					1.00	0.89–1.13	1.01	0.89–1.14
Perinatal death								
Crude Model	1.03	0.79–1.34	0.83	0.72–0.96	0.81	0.60–1.08	0.81	0.60–1.08
Adjusted Model I	0.97	0.74–1.27	0.98	0.85–1.14	1.13	0.80–1.60	1.13	0.80–1.60
Adjusted Model II					1.00	0.67–1.48	1.00	0.67–1.48

Adjusted Model I- adjusted for socio demographic factors; maternal age, marital status of mothers, area of residence, smoking status and year of child birth

Adjusted Model II- adjusted for number of previous TOPs, gestational age at TOP and the year of last TOP

^1^ No TOP group is used as reference group

^2^ Medical group is used as reference group

^3^ Medical group is used as reference group and includes mothers with only one medical and one surgical TOP

After adjusting for the sociodemographic factors, the mothers with previous medical TOPs had decreased risk for preterm birth and low birth weight when compared to the mothers with no previous TOPs (Table 4). The increased risk for adverse outcomes when comparing the mothers with surgical TOPs to the mothers with no TOPs, did not remain significant, after controlling for background characteristics. However, mothers with surgical TOPs had marginally increased risk for SGA compared to the mothers with no TOPs.

The mothers with previous surgical TOPs had higher risk for preterm birth and newborn with low birth weight than those mothers with previous medical TOPs (Table 4). After an additional adjustment for the number of previous TOPs, the gestational age at the time of TOP, year of the last TOP, risk for preterm birth (<37 weeks) and risk for newborn with low birth weight remained significant (Table 4).

After restricting the analysis to those mothers having had only one previous TOP, and adjusting for confounders, the results did not change: the mothers having had only one previous surgical TOP had increased risks for preterm birth and low birth weight when compared to the mothers with only one previous medical TOP (Table 4).

## Discussion

The first-time mothers with previous TOP were much younger, single and more often smokers than the mothers without previous TOP. In addition, the mothers with previous medical TOPs had a reduced risk for preterm birth and low birth weight when compared with the mothers with no previous TOP. All the poor outcomes measured, with the exception of small for gestational age and perinatal deaths, were more common among the mothers with previous surgical TOPs than among the mothers with previous medical TOPs. This was also true among those mothers who had gone through only one TOP before their first birth.

Our nationwide study covered all the first-time mothers having had singleton births during the time period ranging from 1996 to 2013, and all of the TOPs performed in Finland during the time period ranging from 1983 to 2013. Because our data did not include TOPs before 1983, some women might have been classified wrongly into the “no TOP group”. However, we assume that there are only few such cases and this will thus not affect our results. The quality of the data from the THL and AR is considered to be very high and reliable [19,20], and earlier studies have compared the information in the medical records with the AR and found that 95% of the information matched and 99% data coverage [19,21]. Our large data set enabled us to study the outcomes in different subgroups, based on the method of terminating pregnancy. Even though we were able to study the outcomes by the method we were not able to separate different medical (mifepristone/misoprostol) or surgical methods (dilatation, curettage or vacuum aspiration) because the register do not contain as detailed information. Furthermore, this is an observational study and it cannot provide evidences for causality.

Overall, our findings that the demographic and reproductive profiles of first-time mothers with histories of previous TOPs differed from those of first-time mothers without histories of previous TOPs are in line with previous studies [8,11,22,23]. In the multivariate logistic regression analysis, we were able to adjust for several background variables; however, we could not adjust for the socioeconomic position of the mothers due to incomplete data (data not shown). In Finland, smoking has been found to be a good proxy for the socioeconomic position [24]; therefore, we used urbanity of municipality and mother’s smoking instead.

Previously, it has been reported that undergoing several TOPs before a woman’s first birth correlated with poorer perinatal outcomes in subsequent births [5–8]. However, we compared the mothers with histories of surgical and medical TOPs, but adjusted for the number of previous TOPs. Moreover, we conducted a subgroup analysis of those having had only one surgical or medical TOP. We also adjusted for the gestational age at the time of TOP and the year of the last TOP, which has not been done in previous studies [12,15,17,25].

There are few studies examining the long-term consequences of medical TOPs [14,16]. However, our finding of a reduced risk of preterm births among the mothers with previous medical TOPs, when compared to the mothers with no previous TOP, is consistent with a previous study from China [13]. In addition, some other studies [14,15,17] indirectly support our finding of no increased risk for preterm births among the mothers with history of medical TOPs. Having had a TOP reflects fertility, and this may explain the better outcomes among those mothers having had medical TOPs compared to the mothers without history of TOPs. Poorer outcomes after surgical TOPs might be due to the reason that the medical TOPs cause less physical trauma to the cervix and the less endometrial damage than the surgical TOPs [6,13,26,27].

As in some prior studies, our unadjusted results showed an increased risk for preterm births among those mothers with surgical TOPs, when compared with those mothers having had no prior TOPs [15,17,18,25]. However, in our study, the significance was lost after adjusting for the sociodemographic factors.

Similar to some previous studies [9,15,17,25], we found a higher risk for preterm births among the mothers with previous surgical TOPs, when compared to the mothers with previous medical TOPs. Medical TOPs may cause less harm to the uterus than surgical TOPs, which can result in better birth outcomes later [13,27]. A recent review and meta-analysis from 21 studies also supports our findings with regard to the association between preterm births and surgical TOPs [28]. In contrast, some previous studies have not found an increased risk for preterm births in subsequent births among mothers with previous surgical TOPs, when compared to mothers with previous medical TOPs [11,14,29]. However, some of those studies did not control for potential confounders, and some were based on self-reported TOPs, which may introduce recall bias [13,14].

Contrary to some of the previous research, our study reported an increased risk for low-birth weight among the mothers with previous surgical TOPs, when compared to the mothers with previous medical TOPs [12–14,16]. However, few studies found a positive association between surgical TOPs and the risk of low birth weight [4,9], which might support our findings.

## Conclusion

Perinatal outcomes did not differ among the mothers with surgical TOPs compared to the mothers with no TOPs, while the outcomes were poorer after surgical TOP(s) than after medical TOP(s). It is important to study the effects of the different methods used for terminating pregnancy to determine the safest method. This could be of importance for healthcare professionals in terms of clinical decision making and counselling women seeking termination of pregnancy, with respect to the method used for termination.
