# Correction: Perinatal outcomes after induced termination of pregnancy by methods: A nationwide register-based study of first births in Finland 1996–2013

**DOI:** 10.1371/journal.pone.0190331

**Published:** 2017-12-20

**Authors:** Situ KC, Elina Hemminki, Mika Gissler, Suvi M. Virtanen, Reija Klemetti

There is an error in affiliation 1 for authors Situ KC and Suvi M. Virtanen. Affiliation 1 should be: Faculty of Social Sciences, University of Tampere, Tampere, Finland.
